# Cloning of *fimH* and *fliC* and expression of the fusion protein FimH/FliC from Uropathogenic *Escherichia coli* (UPEC) isolated in Iran

**Published:** 2012-06

**Authors:** Karam MR Asadi, M Oloomi, M Habibi, S Bouzari

**Affiliations:** Department of Molecular Biology, Pasteur Institute of Iran, Tehran, Iran

**Keywords:** urinary tract infection, Uropathogenic *Escherichia coli*, fimH, fliC, fusion protein expression

## Abstract

**Background and Objectives:**

Urinary tract infection (UTI) is one of the most common infections in the world. The majority of UTIs are caused by Uropathogenic *Escherichia coli* (UPEC) strains. FimH and FliC are the most important virulence factors of UPEC. To date, any ideal vaccine against UTI has not been approved for human use and we need to test new targets to develop an ideal vaccine against UTI. In this study, we constructed fusion fimH/fliC of UPEC as a novel vaccine candidate against UTI.

**Material and Methods:**

PCR amplification of *fimH* and *fliC* genes of the UPEC isolates was performed by specific primers designed for this purpose. Construction of fimH/fliC hybrid gene was performed by overlap PCR. The fimH, fliC and fimH/fliC were cloned in pET28a vector. The confirmation of expression of the proteins was done by SDS-PAGE and Western blot.

**Results:**

The *fliC* and *fimH* genes were amplified in all of the UPEC isolates tested. The fimH showed significant homology with the sequences in GenBank. We generated a fusion consisting of the fimH linked to the N-terminal end of fliC. Sequencing of the fusion fimH/fliC showed that fusion was constructed correctly. SDS-PAGE and western blot confirmed the expression of the proteins in optimized condition.

**Conclusion:**

Urinary tract infection is a huge burden on healthcare system in many countries. UPEC is isolated in around 80% of UTI cases. Antibiotic therapy resulted in the emergence of antibiotic resistance in UPEC strains. This is the major cause for an increasing requirement for a vaccine to prevent UTI. This work describes for the first time the construction of a novel fusion protein from Iranian UPEC isolates. Further immunological studies are required for evaluation of this protein as a novel and safe vaccine candidate against UTI caused by UPEC.

## INTRODUCTION

Urinary tract infection (UTI) is one of the most common infections in all age groups that cause significant mortality and morbidity in the world (1, 2). For example, UTI accounts for about 7 million office visits, 1 million emergency room visits and 250,000 hospitalizations in the United States, with an annual cost of $ 2.4 billion (2, 3). The studies show that UTI is one the most important infectious diseases in Iran that needs further consideration (4–7). Approximately, 50% of women experience one UTI in their life time and about 25% of them will have another UTI within 6 months (2). Most of the UTIs occur in the bladder (cystitis), but the infection can lead to serious conditions including pyelonephritis, bacteremia and sometimes death (8, 9). Uropathogenic *Escherichia coli* (UPEC), isolated from 50-90% of all reported UTIs, account for about 70-90% of acute cystitis and 250,000 pyelonephritis cases reported annually in the United States (2, 10).

UPEC have different virulence factors including adhesive fimbriae, toxins, siderophores, flagella and capsule (2, 11). Type1 pilus, by having the adhesin fimH at its tip, is the most important virulence factor of UPEC (12). FimH has critical role in binding and colonization of UPEC to urothelial cells (uroplakins), and invasion of UPEC into the bladder epithelial cells (2, 12). The flagella are composed of polymerized subunits of flagellin encoded by *the fliC* gene (9, 13). Motility by flagella causes ascension of UPEC from bladder into the kidney and contribution of UPEC in efficient colonization of the urinary tract (8, 9, 14, 15).

As mention above, UTI caused by UPEC is one of the most common infections in the world that can lead to serious conditions such as bacteremia and renal scarring (1–3). Furthermore, the prevalence of antimicrobial resistance in patients with UTI is increasing which can complicate future treatment of these infections (2, 16). These are some of the reasons for the need to develop an efficacious vaccine against UTI. Some of the virulence factors of UPEC tested as vaccine targets against UTI are fimH, papG, Dr adhesin and siderophore receptor IroN (16). We need to test new target antigens or other technologies to develop an ideal and safe vaccine against UTI. In this study, we isolated *fimH* and *fliC* genes from Iranian UPEC isolates and constructed recombinant fusion (hybrid) protein FimH/FliC of UPEC as a novel fusion protein that might act as a vaccine candidate against UTIs.

## MATERIALS AND METHODS

### Collection of samples

Twenty two urine samples with significant bacteriuria culture of ≥ 10^5^ colony-forming units (cfu/ml) were collected from several hospitals in Tehran, Iran. All urine samples were cultured on blood agar and MacConkey agar for 24 h at 37°C. Bacterial identification was performed by routine culture method and biochemical tests (11).

### DNA extraction and PCR amplification

All UPEC isolates were grown in 5 ml of Luria Bertani (LB) broth medium. Genomic DNA was extracted using the Phenol & Chloroform method. PCR amplification of *fimH* and *fliC* genes was performed by primers designed for conserved 3' and 5' end of the genes, the primers are listed in Table 1. These primers were designed based on the *fimH* gene of UTI89 strain (GenBank accession nos****. NC_007946.1) and the *fliC* gene of CFT073 strain (GenBank accession nos. NC_004431.1). PCR reactions were performed by eppendorf thermocycler, PCRs were carried out in 50 µl volume containing 2 µl of DNA template, 5 µl of 10x reaction buffer, 2 µl of dNTPs (10 mM), 2 µl of Mgcl2 (50 mM), 2 µl of each primer (10 pmol), and 1U of pfu DNA polymerase (fermentas). The PCR condition for amplification of *fimH* gene included initial denaturation for 5 min at 94°C, followed by 10 cycles, each consisting of 60 s at 94°C, 60 s at 45°C, and 60 s at 72°C, and then 20 cycles, each consisting of 60 s at 94°C, 60 s at 55°C, and 60 s at 72°C, with a final extension at 72°C for 5 min. The PCR condition for amplification of *fliC* gene was similar to *fimH* gene but we used 50°C instead of 45°C in annealing step. After amplification of *fimH* and *fliC* genes, 5 µl of samples were subjected to electrophoresis on a 1% agarose gel to confirm the presence of the amplified products.

**Table 1:** Characteristics of primers used in this study.

Number	Primer Name	Sequence (5′-3′)	Tm (°C)	Reference
1	FimH-For	CATGCCATGGCCATGAAACGAGTTATTACC	66.8	This study
2	FimH-Rev	CCCAAGCTTTTGATAAACAAAAGTCAC	63.9	This study
3	FliC-For	CATGCCATGGCGATGGCACAAGTCATTAAT	66.8	This study
4	FliC-Rev	CCCAAGCTTACCCTGCAGCAGAGACAG	63.3	This study
5	Fusion-For	ACTTTTGTTTATCAAATGGCACAAGTCATT	59.9	This study
6	Fusion-Rev	AATGACTTGTGCCATTTGATAAACAAAAGT	59.9	This study
7	Fm-For	CCAGCGAATAACACGGTATC	56	This study
8	Fl-Rev	AACGGTTAGCAATCGCCTGACC	60	This study

### Cloning of genes into the T/A cloning vector

The PCR products of *fimH* and *fliC* genes were purified by the use of gel extraction kit (Roche) and ligated into the pTZ57R (MBI Fermentas) cloning vector. T4 DNA ligase (Fermentas) was used for the ligation. The resulting plasmids were transformed into competent *E. coli* (Top10) (invitrogen) by following the manufacturer's instructions. The MacConkey plates containing 100µg/µl ampicillin were used for selection of transformed colonies. The white colonies were selected and following an overnight cultivation, were subjected to plasmid extraction and PCR. In order to verify the fidelity of the cloned fragments, the selected recombinant plasmids were subjected to sequencing (MWG service).

### Construction of fimH/fliC hybrid gene

**Fig. 1:**
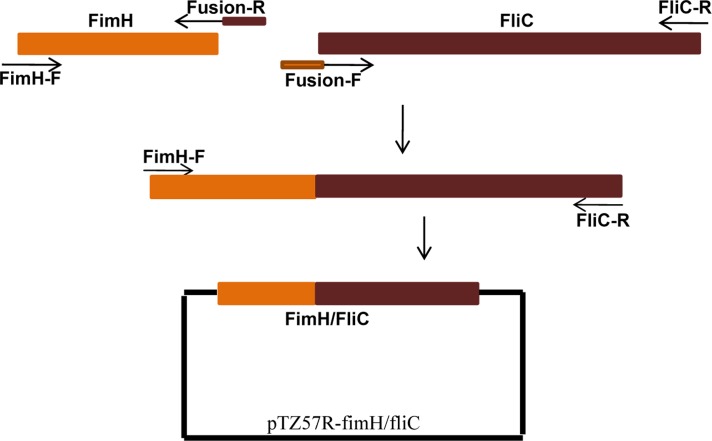
Method used for fusion of two genes. The fused product is then cloned into pTZ57R vector.

For construction of hybrid fimH/fliC, a *fimH* and *fliC* genes, with the highest sequences similarity to *fimH* of UTI89 and *fliC* of CFT057 strains were selected. The schematic presentation of construction of the fusion is shown in Fig. 1. At first, amplification of *fimH* gene was done by primers 1 and 6 and then amplification of *fliC* gene performed by primers 4 and 5. The sequences of primers used for amplification and fusion of the fimH and fliC are shown in Table 1. After gel extraction of the PCR products, the purified products were mixed together and used as template at final PCR reaction for construction of fusion genes. The final PCR reaction was done by the use of primers 1 and 4. The amplified fused fragments were cloned in pTZ57R vector (schematic in Fig. 1) and the selected recombinant plasmids were subjected to sequencing (MWG service). Sequencing of the fusion gene was performed by using designed internal (primers FimH-For, FliC-Rev and Fm-For, Fl-Rev) and universal primers. The sequence of primers used for sequencing of the fusion gene is shown in Table 1.

### Expression of the fused genes

The *fimH*, *fliC* and *fimH/fliC* genes cloned in pTZ57R vector were used as the source of DNA for cloning into expression vector pET28a (Novagen). PCRs were performed using the Pfu DNA polymerase (fermentas) and primers designed to introduce *Nco*I site at the 5′-terminus and a *Hind*III site at 3′-terminus of the genes. After amplification, the PCR products were gel-purified and digested with the *Nco*I and *Hind*III restriction enzymes. The digested products were cloned into the *Nco*I and *Hind*III sites of expression vector pET28a under the T7 promotor with histidine Tag (his6) to generate proteins with His6 at the C-terminal of the protein. The ligated plasmid was transformed into competent *E. coli* BL21 (DE3). The selected clones were analyzed by gel electrophoresis, PCR, restriction analysis and sequencing. Recombinant *E. coli* BL21 (DE3) cells were grown overnight in Luria broth (LB) medium containing kanamycin (50 µg/ml) at 37°C. On the following day, 500 ml of LB medium was inoculated with 5 ml of the overnight culture of BL21 (DE3). The inoculated culture was grown with agitation under aerobic conditions at 37°C. Then, expression of the cloned gene was induced by different concentrations of IPTG (final concentration 0.1 mM-1 mM). After incubation for 4 h, cells were harvested by centrifugation at 4°C and were stored at -20°C till further use.

### SDS-PAGE and Western blot

For analysis of expression of proteins, the Sodium Dodecyl Sulfate-Polyacrylamide Gel Electrophoresis (SDS-PAGE) was used. The bacterial pellets were suspend in loading buffer, heated for 5 min at 95°C and 30 µl of each sample was subjected to 12-15% SDS-PAGE gel. For western blot, the samples were separated by SDS-PAGE and transferred in nitrocellulose membrane (Schleicher and Schuell) using a liquid transfer system (Bio-Rad). Membranes were blocked with skimmed milk in PBST (PBS 1% + Tween 20) and then washed several times with PBST. The membranes were incubated with the conjugated His–tag antibody (invitrogen) for 2 h at room temperature. After washing with PBST, HRP conjugated antibody was used as secondary antibody.

## RESULTS

**Fig. 2:**
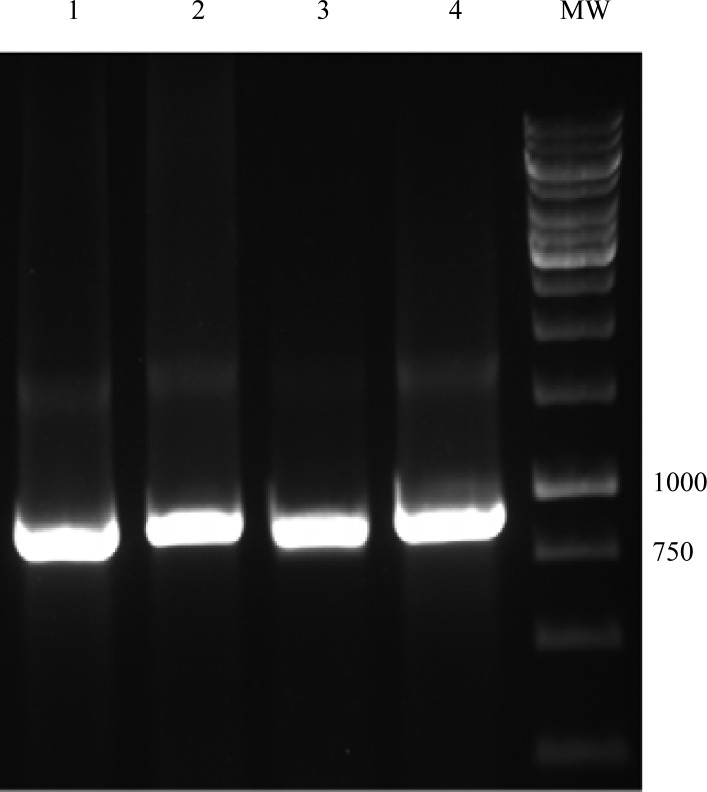
PCR amplification of *fimH* gene. Lane 1-4: products of *fimH* gene (900 bp); MW: Molecular weight marker (1 kb ladder DNA).

**Fig. 3:**
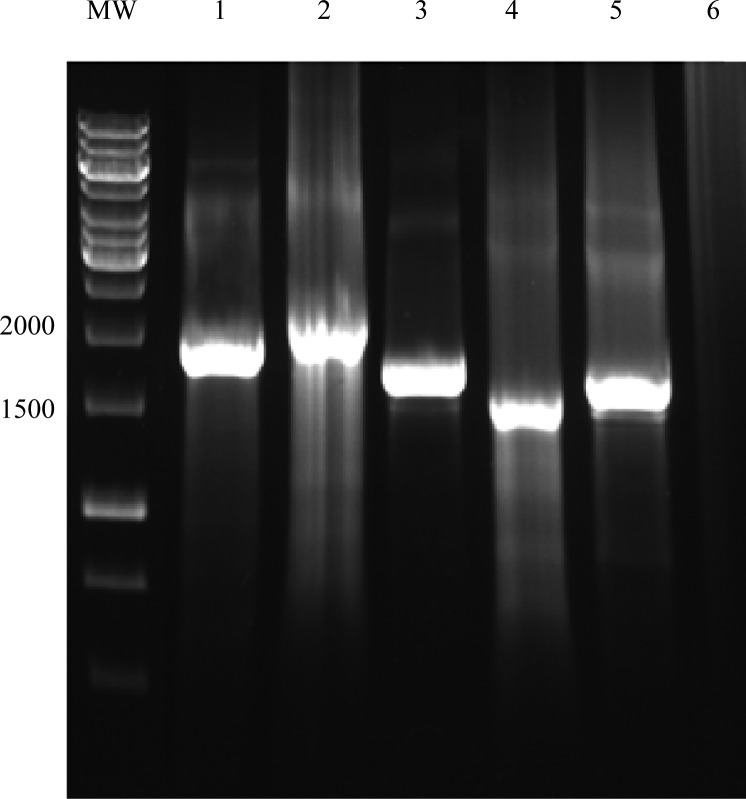
PCR amplification of *fliC* gene. Lane 1-5: products of *fliC* gene (about 1500-2000 bp); Lane 6: Negative control; MW: Molecular weight marker.

Twenty *Escherichia coli* isolates from 22 urine samples were detected. The chromosomal DNA of the *E. coli* isolates was extracted and used to perform PCR assay. The PCR conditions were optimized for amplification of *fimH* and *fliC* genes. The *fliC* and *fimH* genes were present in all of the *E. coli* isolates. Electrophoresis of PCR products showed that the length of PCR fragment of *fimH* genes was approximately 900 bp (Fig. 2) and bands ranging from about 1500 to 2000 bp for *fliC* gene (Fig. 3) were obtained in the isolates tested. Comparison of the sequences of *fimH* and *fliC* genes with sequences of the genes in the GenBank showed significant homology (≥98%) for the fimH from our *E. coli* isolates and fimH from UPEC strain in GenBank. However, the sequence of *fliC* genes showed different homology ranging from 50 to 98% to sequence of fliC from UPEC strain in the GenBank.

**Fig. 4:**
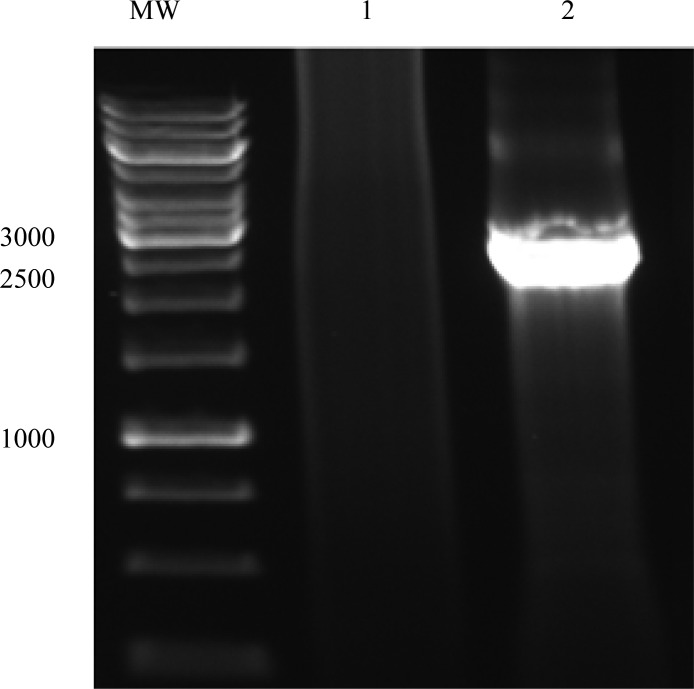
Construction of fusion *fimH/fliC* gene. Lane 1: Negative control; Lane 2: fusion *fimH/fliC* gene (about 2600 bp); MW: Molecular weight marker.

**Fig. 5:**
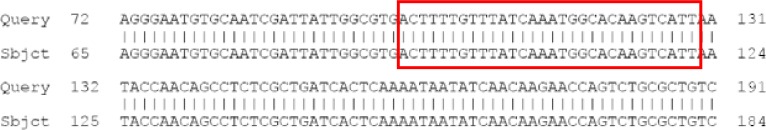
Nucleotide sequence of fused part of fimH/fliC.

After comparing of fimH and fliC sequences with those published in GenBank using BLAST (www.ncbi.nih.gov), we selected an *E. coli* isolate with highest homology in *fimH* and *fliC* genes with sequence of these genes in GenBank to construct the fusion protein. We generated a fusion consisting of the *fimH* gene linked to the N-terminal end of *fliC* gene (Fig. 4) by the use of overlap PCR method (Fig. 1). Sequencing of the fusion fimH/fliC by internal and universal primers showed that fusion was constructed precisely (Fig. 5).

**Fig. 6:**
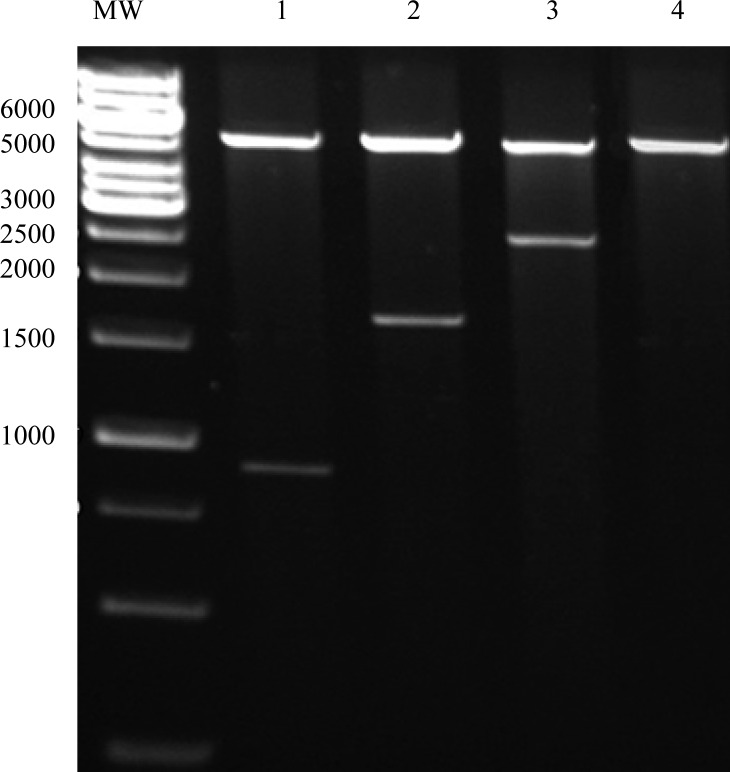
Digestion of genes cloned in pET28a vector by *Nco*I-*Hind*III. MW: Molecular weight marker; Lane 1: Digested pET-fimH; Lane 2: Digested pET-fliC; Lane 3: Digested pET-fimH/fliC; Lane 4: pET28a Undigested.

After amplification of *fimH*, *fliC* and fusion genes in pTZ57R vector and digestion of eluted fragment by *Nco*I and *Hind*III enzymes, they were cloned in pET28a and transformed into the *E. coli* BL21 (DE3). Confirmation of cloning of the genes by digestion with *Nco*I and *Hind*III restriction enzymes is shown in Fig. 6.

**Fig. 7:**
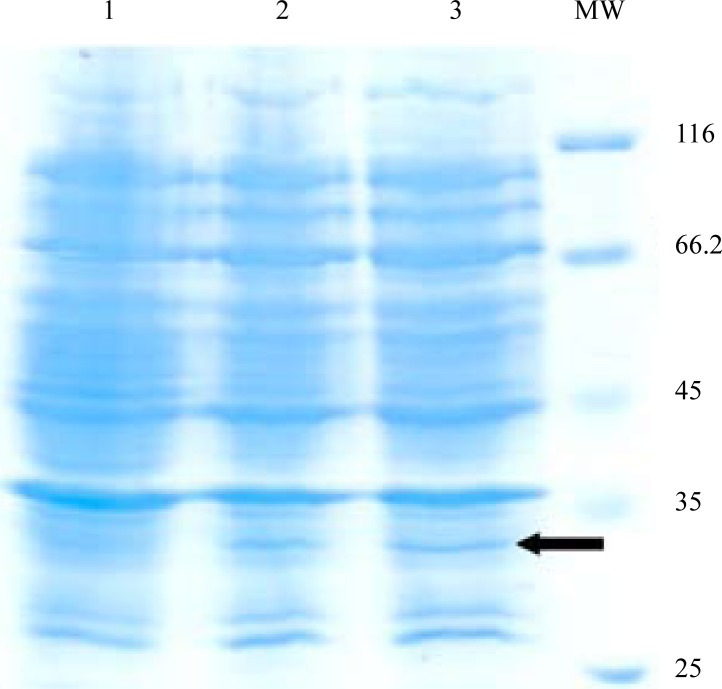
Analysis of expressed products of fimH by% 15 SDS-PAGE. Lane 1: Uninduced construct; Lane 2: pET-fimH induced by IPTG 0.5 mM; Lane 3: pET-fimH induced by IPTG 1 mM; MW: protein marker.

**Fig. 8:**
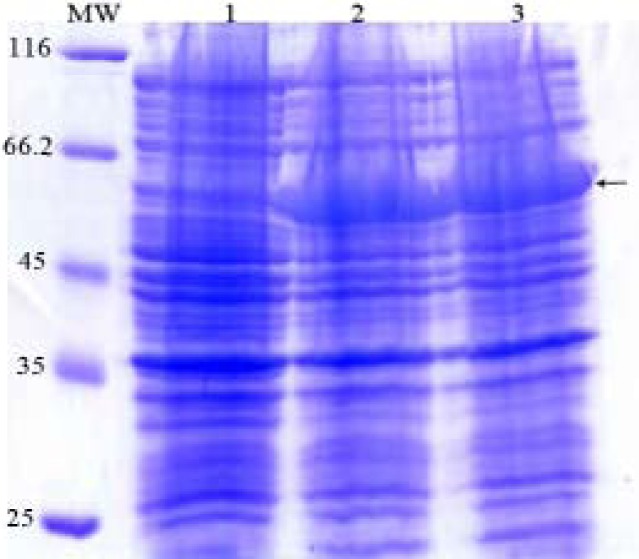
Analysis of expressed products of fliC gene by% 15 SDS-PAGE. Lane 1: uninduced construct; Lane 2: pET-fliC induced by IPTG 0.5 mM; Lane 3: pET-fliC induced by IPTG 1 mM; MW: protein marker.

**Fig. 9:**
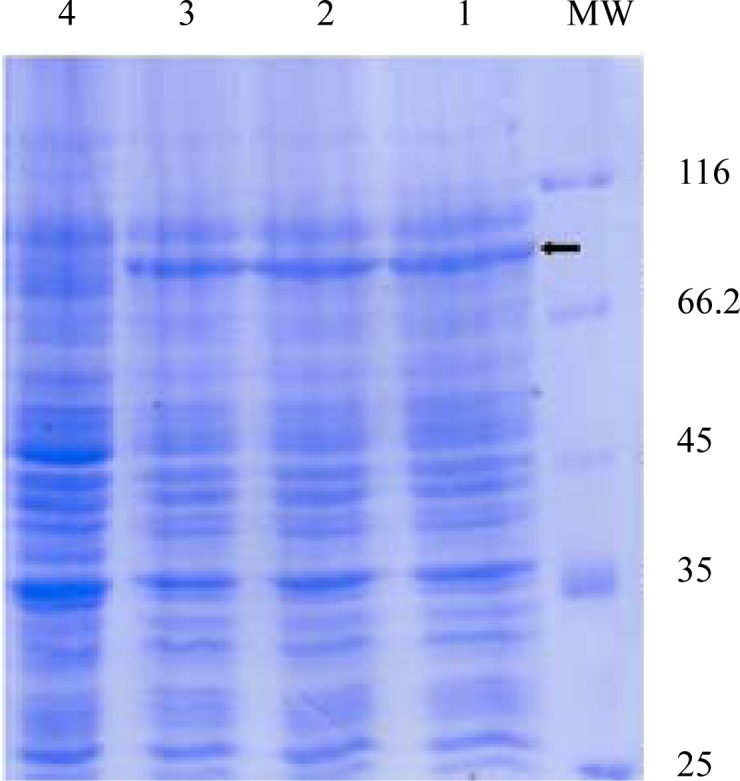
Analysis of expressed products of fusion gene by %12 SDS-PAGE. Lane 1: pET-fusion induced by IPTG 0.2 mM; Lane 2: pET-fusion induced by IPTG 0.5 mM; Lane 3: pET-fusion induced by IPTG 1 mM; Lane 4: uninduced construct; MW: protein marker.

**Fig. 10:**
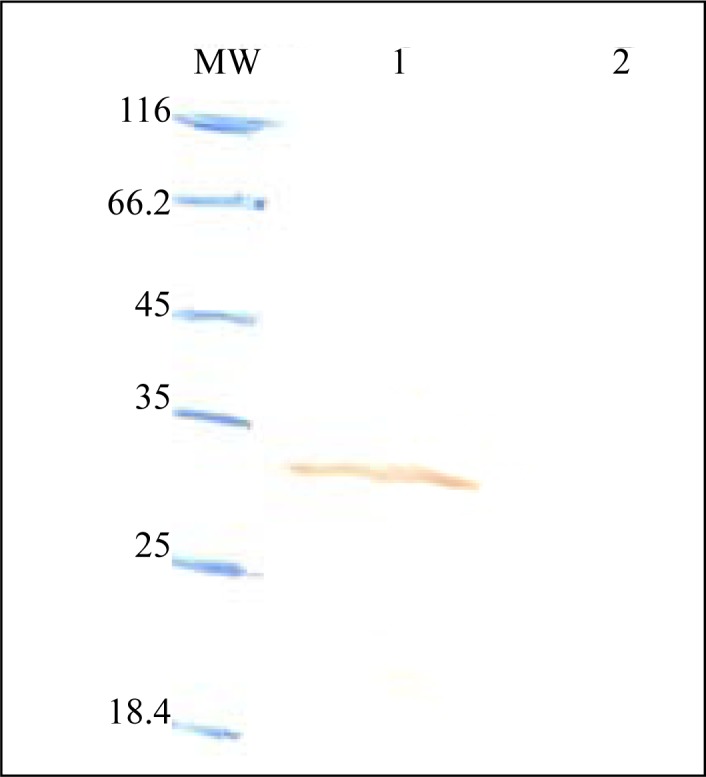
Western blot analysis of FimH. Lane 1: pET-fimH induced by IPTG 0.5 mM; Lane 2; uninduced construct; MW: protein marker.

**Fig. 11:**
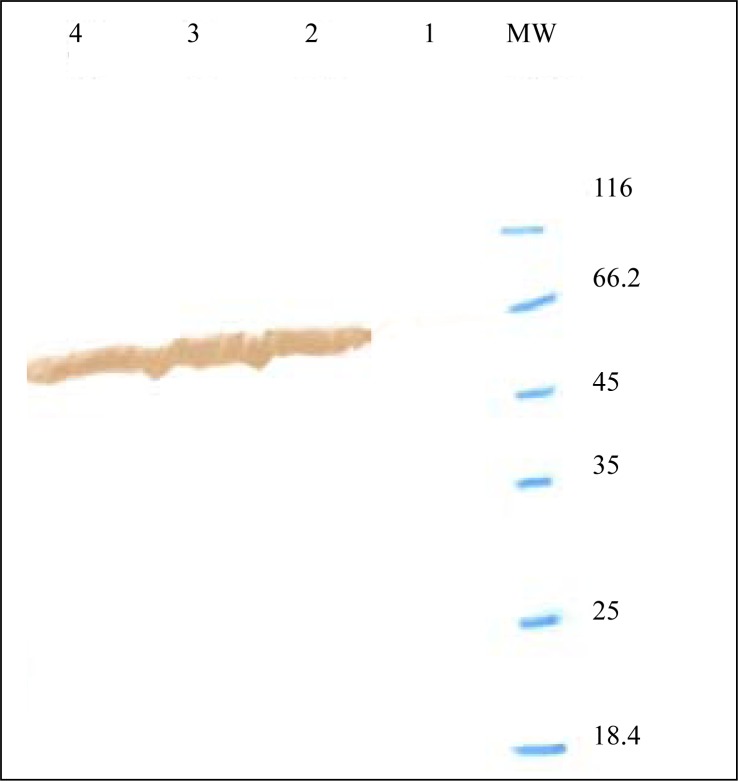
Western blot analysis of FliC. Lane 1: uninduced construct; Lane 2: pET-fliC induced by IPTG 0.5 mM; Lane 3: pET-fliC induced by IPTG 1 mM; Lane 4: pET-fliC induced by IPTG 0.2 mM; MW: protein marker.

**Fig. 12:**
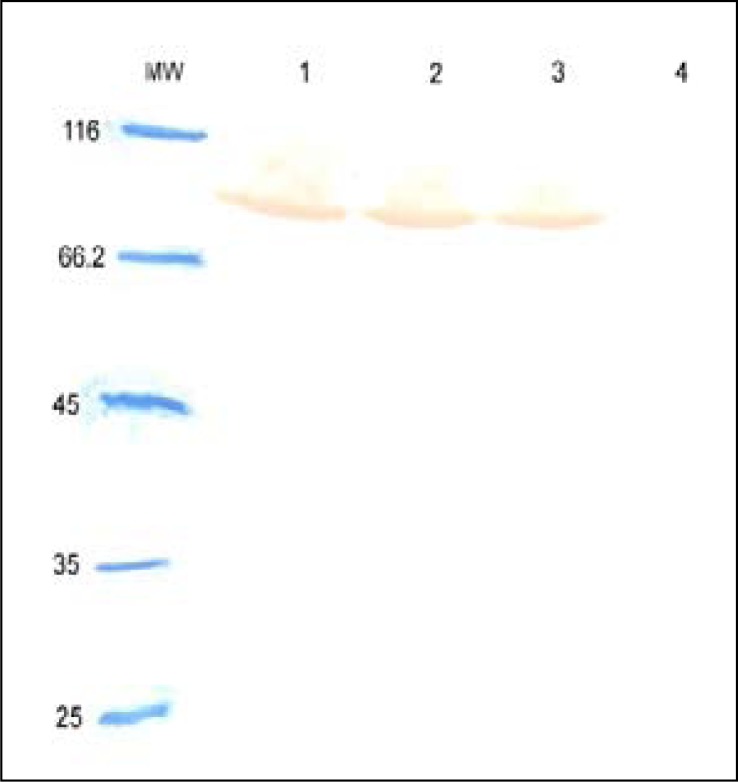
Western blot analysis of fusion. Lane 1: pET-fusion induced by IPTG 0.2 mM; Lane 2: pET-fusion induced by IPTG 0.5 mM; Lane 3: pET-fusion induced by IPTG 1 mM; Lane 4: uninduced construct; MW: protein marker.

Expression of *fimH, fliC* and fusion genes cloned in pET28a optimized by parameters such as different concentration of IPTG and incubation time. Optimum expression was obtained with IPTG 0.5 mM and incubation time of 4 h for FimH, IPTG 0.2 mM and incubation time of 4 h for FliC and IPTG 0.2 mM with incubation time of 5 h for fusion. The level of expression and purity of FimH, FliC and fusion protein were analyzed by SDS-PAGE then by western blotting using anti-His antibody. The results of SDS-PAGE of the FimH, FliC and fusion proteins are shown in Figs. 7, 8 and 9, respectively. The results of Western blot of the FimH, FliC and fusion proteins are shown in Figure 10, 11 and 12, respectively. The analysis of SDS-PAGE and western blot of the proteins showed single bands at the size of approximately 30, 60 and 90 KDa for FimH, FliC and fusion protein, respectively.

## DISCUSSION

Urinary tract infection (UTI) is one of the most common infections diagnosed in outpatients and inpatients (1, 2). Uropathogenic *Escherichia coli* (UPEC) are the most common pathogen found in urinary tract infection, being isolated in around 80% of UTI cases (2, 10). Our study showed high rate of UTI by UPEC among patients of UTI. In other studies from Iran the isolation rate of UPEC was 57.4% and 74.6% (4, 17). The emergence of antibiotic resistance in UPEC strains in the world is the major cause for an increasing requirement for vaccine development against UTI (2, 16).

One of the important criteria for an ideal vaccine target against UPEC is its wide distribution among clinical UPEC isolates. The studies showed that fimH is highly conserved among UPEC strains (3, 12). For example, Tiba et al. studied a total of 162 UPEC strains from patients with cystitis and found fimH in 97.5% of strains (11). In the study of Johnson et al (18), the prevalence of fimH was similar to our results. Flagellin (fliC) is another conserved gene among UPEC strains, about 70% of clinical isolates obtained from patients with UTI harbored *fliC* gene (8). In our study, all of the isolated *E. coli* from patients showed presence of the gene.

We selected the pET28a expression system for expression of fimH, fliC and fusion fimH/fliC protein. The T7 promoter of the expression system results in robust expression of the target gene. After optimization of expression, the yields of expression of the recombinant proteins were 7 mg, 3 mg and 1 mg per liter of bacterial cultures for FliC, fusion and FimH proteins, respectively. The results indicated that the quantity of expressed FimH in comparison with FliC and fusion is low. There are multiple parameters that can be varied when optimizing an expression protocol, from selecting a vector with the appropriate promoter, to choosing an appropriate induction temperature (19, 20). Initially, we used pBAD expression vector by adding different concentration of inducer L-arabinose (0.002-20%) (Data not shown).

The pBAD expression vector has extremely low basal expression, and by having a signal sequence expresses proteins in periplasm (20). Although, we changed expression conditions including temperature (15-37°C), media, concentration of the inducer and incubation time, the expression yield of FimH protein was even lower than the yield of the protein in pET expression system. In a similar result, the expression yield of FliC and fusion proteins in pBAD were lower than the yield of the proteins in pET expression system. The properties of the gene and/or protein sequence can influence expression of the proteins in *E.coli* host (19, 20).

One of the most common reasons that proteins fail to express in *E. coli* is the presence of rare codons in the gene of interest (19). The genes that contain significant numbers of rare codons are more likely to completely fail to express or express at low levels (19, 21). We quantified the number of rare codons in *fimH, fliC* and their fusion http://nihserver.mbi.ucla.edu/RACC/. The numbers of rare codons were 6, 1 and 7 for fimH, fliC and their fusion, respectively. Other reason for low yield of FimH protein could be the absence of FimC protein (19–21). Pili type1 encoded protein namely FimC that act as a chaperone molecule for FimH (22, 23). FimC interacts with the FimH and stabilized the FimH subunit in the periplasm and prevent the adhesin from aggregation and proteolytic degradation (22, 23). Thus, in the absence of the chaperone, the FimH subunit can be degraded and the yield of FimH in the host decreased (22, 23).

In conclusion, this work describes, for the first time, the construction of a genetically constructed FimH/FliC protein from Iranian UPEC isolates. Hence further immunological studies are required for evaluation of this protein as a novel and safe vaccine candidate against UTI caused by UPEC.
